# Joint Modulation of Postural and Neural Correlates in Response to Motivational Images in Non-Clinical Drinkers

**DOI:** 10.3390/biology14091172

**Published:** 2025-09-02

**Authors:** Amel Zitouni, Mbarka Akounach, Sumeyye Kızılışık, Salvatore Campanella, Ardalan Aarabi, Thierry Lelard, Harold Mouras

**Affiliations:** 1 UR-UPJV 4559, Functional and Pathological Neurosciences Laboratory, Health Research Universitary Center, Medecine UFR, Picardy Jules Verne University, CEDEX, 80054 Amiens, France; amel05zitouni@gmail.com (A.Z.); mbarka.akounach@etud.u-picardie.fr (M.A.); sumeyye.kizilisik@gmail.com (S.K.); ardalan.aarabi@u-picardie.fr (A.A.); 2 Laboratoire de Psychologie Medicale et d’Addictologie, ULB Neuroscience Institute (UNI), CHU Brugmann-Universite Libre de Bruxelles (U.L.B.), 1020 Bruxelles, Belgium; salvatore.campanella@chu-brugmann.be; 3 UR-UPJV 3300, Physiological Adaptations to Exercise and Exercise Rehabilitation, Sport Sciences Department, Picardy Jules Verne University, 80025 Amiens, France; thierry.lelard@u-picardie.fr

**Keywords:** EEG, incentives, alcohol, food, neural ERPs

## Abstract

Approach or avoidance behaviors toward appetitive stimuli, such as alcohol and food, reflect the engagement of motivational states that are fundamental to adaptation of human behavior. Investigating early motor or neural responses to these stimuli provides valuable insights into the underlying mechanisms of these behaviors. This study employed an integrative approach combining postural and electrophysiological measures to explore the impact of alcohol consumption levels on early postural and neural responses to visual alcohol and food stimuli. The objective was to identify early automatic markers of approach or avoidance, and to examine correlations between motor and neural responses. Forty-six participants were divided into two groups (“Low” and “High”) according to their level of alcohol consumption (AUDIT scores). They were exposed to images of alcoholic beverages, non-alcoholic beverages, and appetitive or neutral foods. Postural responses were recorded using a force platform, and brain activity was measured via EEG. Displacement of the center of pressure along the anteroposterior axis, as well as the P100 and N100 components, were analyzed. “High” participants exhibited greater anterior postural displacement in response to alcohol during the first two seconds of stimulus exposure. In contrast, “Low” participants showed early avoidance responses. Significant correlations were found between event-related potential (ERP) wave latencies and postural displacement during the first second of exposure to alcohol-related stimuli. AUDIT scores were also positively correlated with early postural displacement and N100 latency following the viewing of alcoholic beverage images. Early perceptual and motor responses are modulated by alcohol consumption habits. These findings support the value of integrative EEG–posture approaches for identifying implicit motivational markers.

## Simple Summary

Images that carry motivational value, such as food or drink, can rapidly capture attention and trigger bodily adjustments, often outside of conscious awareness. These early reactions may play an important role in guiding everyday behavior. In this study, we combined two measures to explore how young adults respond to such images: subtle body movements recorded through posture, and rapid brain activity recorded through electrical signals at the scalp. Across participants, we found that motivational images produced both measurable shifts in posture and distinct brain responses within the first fractions of a second. This shows that perception and action are closely linked from the very beginning of processing. As an additional step, we compared individuals with different drinking habits. Those with lighter alcohol use displayed stronger and earlier brain responses and more avoidance-like posture, while heavier drinkers showed weaker brain signals and a slight forward movement toward alcohol cues. Overall, this research demonstrates that combining brain and body measures can provide new insights into how motivational cues influence behavior.

## 1. Introduction

Motivated behaviors, according to Stuber [1], can be defined as the set of approach, avoidance, or action behaviors in response to specific sensory stimuli in order to satisfy a biological need. Motivated behaviors are organized around two fundamental systems, as described by Lang [2]: one appetitive, the other defensive. The appetitive system is associated with approach behaviors directed toward positive events, while the defensive system is involved in withdrawal behaviors aimed at avoiding negative events [2,3]. These two systems are regulated by a complex network of brain circuits involving structures such as the amygdala, the nucleus accumbens, the orbitofrontal cortex, and the anterior cingulate cortex [4]. Among the stimuli perceived as appetitive, food and alcohol are notable for their strong capacity to activate reward circuits. Indeed, it has been established that synaptic modifications within the mesolimbic system are associated with the rewarding effects of psychostimulants such as alcohol, but also with those of natural rewards, such as food [5].

In recent years, posturography has proven to be a valuable tool for exploring motor responses to emotional stimuli, thus offering an innovative approach to studying the mechanisms of approach and avoidance. The study of the interaction between motor and emotional processes, theorized by Darwin in 1872, suggests that emotional state influences postural control [6]. Indeed, several studies investigating the relationship between emotions and the motor system have analyzed postural variations in response to emotional stimuli using posturography [7,8,9]. These studies described the body’s postural oscillations based on the displacement of the center of pressure (COP) measured by posturographic platforms. Posturography allows for the measurement of movement variations in the anteroposterior (COPy) and mediolateral (COPx) directions. The biphasic theory of emotion suggests that when subjects stood on a force platform while viewing images with positive or negative emotional valence, pleasant stimuli elicited a forward body sway (“approach”), whereas unpleasant stimuli resulted in a backward sway (“avoidance”) [10]. In some cases, these unpleasant stimuli could also induce a phenomenon of postural “freezing” [11].

To date, very few studies have used posturography to assess postural responses to visual stimuli related to alcohol or food. Among these studies, Noel with colleagues showed that patients with Alcohol Use Disorder (AUD) who relapsed at the end of treatment exhibited a significant tendency to lean backward (avoidance) in the first seconds of exposure to alcohol images, compared to those who remained abstinent [12]. In the context of food stimuli, Brunyé with colleagues found that participants tended to lean toward images of foods they preferred, while they leaned away from images of foods they disliked [13]. The use of posturography in response to the presentation of motivational stimuli, and particularly in the context of alcohol and food, remains understudied. Research in this area is therefore still limited and requires further investigation to better understand how stimuli related to motivated behaviors can modulate postural control.

Most studies investigating individuals’ automatic tendencies to approach or avoid alcohol or food-related stimuli have employed tasks such as the Approach–Avoidance Task (AAT) [14] or the Stimulus Response Compatibility (SRC) Task [15]. Several studies using these paradigms showed that non-clinical individuals with high levels of alcohol consumption, as well as AUD patients, exhibit automatic approach behaviors toward alcohol-related cues [16,17]. Barkby with colleagues demonstrated that, in AUD patients, there was a positive correlation between their alcohol consumption prior to treatment and their automatic approach tendencies toward alcohol-related stimuli [18]. For food, other research [19,20] has identified automatic approach tendencies toward appetitive food stimuli in individuals undergoing dietary restriction, along with increased attentional bias in individuals characterized by “external” eating behavior, defined as eating in response to environmental cues rather than internal hunger signals. However, these tasks involve instructed and controlled movements (e.g., joystick movements or button presses) that may not fully reflect the spontaneous bodily reactions that occur when individuals are exposed to motivationally relevant stimuli. In contrast, posturography allows to capture automatic changes in posture, offering a more direct way to assess approach–avoidance tendencies.

Electroencephalography (EEG) is a non-invasive and painless method that continuously records brain activity using electrodes placed on the scalp. Although it has limited spatial resolution and is sensitive to noise, EEG allows for the investigation of the cerebral organization of cognitive processes with high temporal resolution. According to Robinson and Berridge, chronic alcohol use induces a sensitization of the appetitive system combined with increased salience of alcohol-related cues [21]. Most studies using event-related potentials (ERP) in the context of alcohol consumption have focused on the P300 component, often considered as a neurobiological marker associated with excessive alcohol intake [22]. For example, Oddy and Barry demonstrated that heavy social drinkers exhibit reduced P300 amplitude during inhibition trials compared to moderate drinkers, suggesting impaired inhibitory control in these populations [23]. Conversely, in alcohol-dependent patients, several studies have reported increased P300 amplitude in response to alcohol-related stimuli compared to neutral stimuli [24,25]. This hyperreactivity to alcohol cues is not exclusive to dependent patients; indeed, Herrmann also observed significantly higher P300 amplitude in response to alcohol-related images compared to neutral images in heavy social drinkers [26]. Abnormalities in the N200 component have also been identified. For instance, Baguley with colleagues reported latency deficits in heavy social drinkers compared to controls [27]. Furthermore, Watson with colleagues showed that higher frequency of alcohol consumption was associated with greater N200 amplitude during inhibition trials in response to alcohol images, which may indicate increased cognitive resource allocation to inhibit responses to alcohol cues [28]. Few studies have examined early components related to perceptual processing, such as P100 and N100, in the context of alcohol consumption. Nevertheless, exploring these components could help elucidate the origins of cognitive alterations observed in later components, such as N200 and P300, among heavy drinkers and AUD patients. A study by Petit with colleagues found greater P100 amplitudes for alcohol-related stimuli compared to neutral stimuli in young binge drinkers, suggesting enhanced perceptual processing of alcohol cues during a go/no-go task [29]. Clinically, substance use disorder appears to result in delayed latency [30,31,32] and reduced amplitude [32] of this component following the presentation of standardized visual stimuli, such as reversed checkerboards and emotional faces. Findings regarding the N100 component reveal notable differences. Herrmann observed that, unlike light drinkers, heavy drinkers exhibited reduced N100 amplitude in response to alcohol-related images compared to neutral images during a passive visual task, where participants were simply exposed to images without any active response required [26]. In contrast, Watson with colleagues, using a go/no-go task, found that participants with more binge drinking episodes had increased N100 amplitudes, regardless of stimulus type (alcoholic or non-alcoholic beverages) [28]. Most research on food stimuli has focused on late components such as P300 and the Late Positive Potential (LPP), which are considered markers of motivated attention. These components generally show higher amplitudes in response to images of appetitive food, particularly in individuals with higher body weight or eating disorders [33]. However, beyond these late components, earlier ones such as P100 and N100 play a crucial role in the study of rapid perceptual responses to food stimuli, though they are often neglected in the literature. Several studies have shown that P100 is modulated by the valence of food stimuli. For example, Schacht with colleagues observed that images of appetitive food induce earlier and larger P100 amplitudes, suggesting rapid and automatic attentional capture [34]. This modulation is interpreted as an indicator of enhanced visual processing of salient stimuli. Similarly, Carbine with colleagues report comparable modulations for the N100, highlighting that these early components may be influenced by factors such as hunger or dietary restriction, indicating that this component reflects individual sensitivity to food cues [33]. While late components such as the P300 and LPP are well established as markers of motivated attention, they reflect relatively controlled and elaborated processing stages. In contrast, earlier components like P100 and N100 index rapid perceptual encoding and automatic attentional orientation. Investigating these early responses is theoretically important, because they may reveal implicit biases toward motivational stimuli before higher-order cognitive regulation comes into play. In this sense, P100 and N100 provide complementary insights to later components, and their joint examination with postural responses allows us to capture the earliest coupling between perception and action in the context of approach–avoidance tendencies.

The primary objective of this study was to simultaneously examine motor responses (using posturography) and early perceptual responses (using event-related potentials, ERPs) to appetitive visual stimuli (alcohol and food) in two groups of participants differing in their level of alcohol consumption, as assessed by the Alcohol Use Disorders Identification Test (AUDIT). By combining behavioral and neurophysiological measures, this research aims to better understand how consumption habits modulate the dynamics of automatic approach or avoidance.

We formulated the following hypotheses: (i) At the postural level, we anticipate that individuals with high AUDIT scores will exhibit a more pronounced approach toward alcohol images, reflected by an anterior displacement of the center of pressure (COP), compared to low consumers. (ii) At the electrophysiological level, we expect that the early components P100 and N100 will be modulated according to both the level of alcohol consumption and the valence of the stimuli. Specifically, images with high motivational value should elicit larger amplitudes. (iii) Finally, we hypothesize a relationship between postural responses and EEG measures, suggesting a functional coupling between early perceptual processing and motor response in the context of motivated behaviors.

Specifically, this study provides the following contributions:

- **A multimodal approach** combining EEG and posturography to examine perceptual–motor responses to appetitive cues.
- **Alcohol-specific effects**, with high consumers showing rapid approach-related posture and delayed early ERP responses, while low consumers exhibited avoidance and enhanced early ERPs.
- **Evidence of perceptual–motor coupling**, as ERP latencies correlated with postural dynamics, even in non-dependent drinkers.

## 2. Materials and Methods

### 2.1. Participants Selection

A total of 65 participants (34 women, 31 men; mean age = 24.98 ± 7.21 years) took part in the study. This sample size was considered sufficient for statistical analyses compared with the numbers generally considered in this type of study, and with the data exclusion criteria inherent in joint EEG–posturography analyses. To take part, volunteers had to meet several conditions: be right-handed, have normal or corrected eyesight, have no diagnosed psychiatric or neurological disorders, have no alcohol consumption problems or eating disorders, and abstain from alcohol in the 24 h prior to experimentation.

To ensure the quality of the data collected, certain exclusions were necessary. Three participants were excluded due to predefined criteria (alcohol consumption prior to the experiment, antidepressant use, and left-handedness), five others due to technical incidents during the experiment, and a further twelve during data preprocessing, due to poor data quality (notably a high rate of trial rejections). As a result, data from 46 participants (23 women; mean age = 25.61 ± 9.77 years and 23 men; mean age = 25.04 ± 4.79 years) were retained for the final analysis. Two groups were defined on the basis of the group’s median AUDIT (Alcohol Use Disorders Test [35]) score (median score = 4): a group of light drinkers (*n* = 24) and a group of heavy drinkers (*n* = 22). All participants signed an informed consent. This study was approved by the Comité d’Éthique pour les Recherches Non Interventionnelles (CERNI, Université de Picardie Jules Verne, Amiens, France) and conducted in accordance with the Declaration of Helsinki (World Medical Association, 2013).

### 2.2. Experimental Paradigm

#### 2.2.1. Stimuli Selection

For the design of the experimental paradigm, four types of stimuli were defined for image presentation: (1) “Alc”: alcoholic beverages, (2) “Food”: palatable foods, (3) “NeutralAlc” and (4) “NeutralFo”. Neutral food was defined as simple foods with no particular color, taste, or presentation (e.g., broccoli, zucchini, hard-boiled eggs, clear soups, etc.). Neutral alcohol” stimuli were defined as non-alcoholic beverages (e.g., water, milk, tea). Stimuli were selected from three recognized databases: CROCUFID (Cross-Cultural Food Image Database, available at: https://osf.io/5jtqx, accessed on 20 March 2024 [36]) for food images, ABPS (Amsterdam Beverage Picture Set, available at https://pmc.ncbi.nlm.nih.gov/articles/PMC5054858 (Appendix S3), accessed on 20 March 2024 [37]), and OzBPS (Australian Beverage Picture Set, available at: https://osf.io/cvwxr, accessed on 20 March 2024 [38]) for alcoholic and non-alcoholic beverages. For more details on the stimuli databases selection procedure, see Supplementary Materials) Several criteria were taken into account when selecting stimuli. Images with a uniform white background were chosen to focus participants’ attention solely on the stimuli. Particular attention was then paid to selecting realistic, natural images, avoiding those featuring artificial elements (e.g., objects without shadows or levitating) that could disrupt natural visual perception. High-resolution and non-pixelated images were preferred. In a small number of cases with excessively bright backgrounds, only the background regions were converted to a neutral grayscale tone using MATLAB 2024b (The MathWorks Inc., Natick, MA, USA) to ensure visual consistency, while the main objects of interest always remained in full color. All images were then normalized to minimize visual differences across categories. A total of 208 images (52 for each of the four conditions) were selected from the identified databases and organized according to the given criteria.

#### 2.2.2. Experimental Procedure

**Figure 1:**
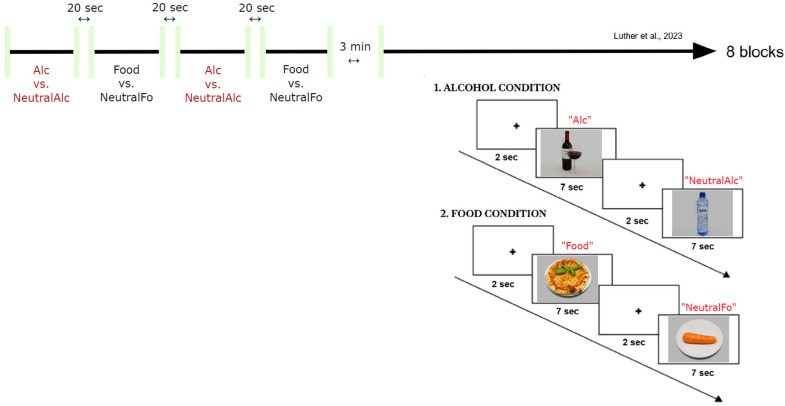
General outline of the experimental paradigm (adapted from Luther with colleagues [39]).

The paradigm of Luther with colleagues, which simultaneously combines EEG and posturography, was adapted for this study [39]. It comprises a total of 208 images, divided as follows: 52 alcohol-related stimuli, 52 alcohol-neutral stimuli, 52 food-related stimuli and 52 food-neutral stimuli. Each stimulus was presented for 7 s (compared with 2 s in the original paradigm by Luther with colleagues [39]), enabling postural changes to be observed, followed by a fixation cross for 2 s. The experiment (Figure 1) was organized in 8 blocks, including 4 food-related blocks and 4 alcohol-related blocks, each block containing 26 images (13 neutral images and 13 appetizing/alcoholic images). There were 20-s breaks between each block, as well as a 3-min break after the fourth block. The entire experiment lasted approximately 35 min.

Participants were recruited for the study via posters, advertisements in local newspapers, and flyers. Once selected, they first received detailed information about the study and signed a consent form in duplicate. To ensure the anonymity of their data, they were assigned a unique seven-character alphanumeric code. After completing the questionnaires, the EEG headset was installed and the participants took their places on the force plate (AMTI, Watertown, MA, USA). E-Prime 3.0 stimulus software (Psychology Software Tools, Inc., Pittsburgh, PA, USA) was used to present the stimuli, AcqKnowledge 5.0 software (BIOPAC Systems, Inc., Goleta, CA, USA) was used for posturography recording, and ActiveTwo 9.02 software for the ActiveTwo EEG system (BioSemi B.V., Amsterdam, The Netherlands) was used for EEG recording. All signals were collected simultaneously. During the experiment, each participant was asked to passively observe the stimuli projected on a screen in front of him/her. At the end of the experiment, participants rated the visualized images along six subjective dimensions: Pleasantness, Unpleasantness, Approach Desire, Consumption Desire, Avoidance Desire, and Intensity on a 9-point scale. Participants responded using a computer keyboard, with no time constraints, but were encouraged to respond quickly. Finally, subjects were compensated 50 euros for their participation.

### 2.3. Measures

#### 2.3.1. Psychometric Data

Prior to the experimental procedure, participants were asked to provide sociodemographic information and to complete a series of validated French-language questionnaires designed to assess various psychological characteristics and behavioral habits.

The Mini-Mental State Examination (MMSE) [40], administered interactively, was used to assess cognitive functions such as thinking, communication, comprehension, and memory.

The Beck Depression Inventory-II (BDI-II) [41], a widely used self-report questionnaire for measuring depressive symptoms and their severity (0–13: No depression, 14–19: Mild depression, 20–28: Moderate depression, 29–63: Severe depression).

The State-Trait Anxiety Inventory (STAI-TRAIT) [42], a 20-item scale, was used to assess participants’ anxiety levels.

The Dutch Eating Behavior Questionnaire (DEBQ) [43] was used to evaluate three different eating behaviors in adults: emotional eating, external eating, and restrained eating.

The Alcohol Use Disorders Identification Test (AUDIT) [35], a questionnaire designed to diagnose excessive alcohol consumption or alcohol use disorders. To distinguish between different levels of alcohol consumption, participants were divided into two groups: low consumers (AUDIT ≤ 4) and high consumers (AUDIT > 4), based on the median (median = 4) of the total AUDIT scores.

Manual dominance was determined using the Edinburgh Handedness Inventory [44].

The Fagerström Test [45] was used to assess tobacco and nicotine addiction.

The characteristics of the study groups are summarized in Table 1.

#### 2.3.2. Posturographic Data

The analog force (Fx, Fy and Fz) and moment (Mx, My, Mz) signals of the force plate (AMTI, Watertown, MA, USA) were acquired at a frequency of 1000 Hz via the MP150 system using AcqKnowledge 5.0 software (BIOPAC Systems, Inc., Goleta, CA, USA). The voltage signals (in Volts) were converted into force values (in Newtons) using the following equation (Equation (1)):

(1) $$F\;=\;\frac{V_{out}\;\times \;10^{3}}{Analog\;sensitivity}.$$

where *F* is the force (in Newtons), *V_out_* is the measured voltage signal (in Volts), and *S* is the analog sensitivity constant (in V/N) provided by the force platform (AMTI, Watertown, MA, USA).

The analog sensitivity values used for this conversion were obtained from the default parameters provided by the force platform (AMTI, Watertown, MA, USA). Posturographic data were low-pass filtered at 5 Hz using a second-order Butterworth filter in MATLAB R2024b (The MathWorks Inc., Natick, MA, USA) in order to remove high-frequency noise while preserving the essential components of the signal. Then, the filtered data were down-sampled to 100 Hz for further analysis. For each image type in every trial, we calculated the position of the Center of Pressure (COP) along the anteroposterior (AP) axis, using the following formula (Equation (2)):

(2) $${COP}_{y}\;=\;\frac{M_{x}}{F_{z}}$$

where *COP_y_* is the anteroposterior displacement of the center of pressure (in meters), *M_x_* is the moment around the x-axis (in Newton·meters), and *F_z_* is the vertical component of the resultant force (in Newtons). These equations are taken from the biomechanical platform installation manual (version 4.4, April 2017; AMTI, Watertown, MA, USA).

#### 2.3.3. EEG Data

EEG data were acquired using the ActiveTwo EEG system (BioSemi B.V., Amsterdam, The Netherlands) with a sampling rate of 1024 Hz. Sixty-four electrodes were placed according to the international 10/20 system. Data preprocessing was performed using the Fieldtrip toolbox (version 20240110, http://www.fieldtriptoolbox.org/download, accessed on 10 March 2025). Ref. [46] integrated into MATLAB 2024b (The MathWorks Inc., Natick, MA, USA). Continuous EEG signals were segmented into epochs of 7.5 s, starting 0.5 s before stimulus onset (baseline) and continuing for 7 s after stimulus presentation. A second-order Butterworth band-pass filter in MATLAB R2024b (The MathWorks Inc., Natick, MA, USA) between 1 and 100 Hz was applied, and artifacts related to line noise were removed using a 50 Hz notch filter. All data were resampled to 512 Hz. To ensure signal quality, defective channels were identified and removed by visual inspection, based on z-score, kurtosis, and variance. Subsequently, independent component analysis (ICA) was performed to identify and remove artifact components, such as those related to ocular, cardiac, or muscular activity. After this step, spherical interpolation was conducted to restore rejected channels using information from neighboring electrodes in order to preserve the spatial integrity of the EEG data, and an average re-reference was applied. Once these cleaning steps were completed, bad trials were visually rejected based on z-score, kurtosis (measuring the concentration of values around the mean), and variance. At the end of preprocessing, a comprehensive visual inspection of the entire signal was carried out to ensure data quality before ERP analysis. The cleaned dataset was then divided into four experimental conditions for further analysis. To ensure data quality, the method proposed by Luther with colleagues [39] was used as a reference. The number of rejected trials per participant was analyzed, and an exclusion threshold was set based on the mean plus one standard deviation (mean + standard deviation). Participants whose number of rejected trials exceeded this threshold were excluded from the study to preserve the overall quality of the analysis. According to this approach, 10 participants were excluded from the analysis.

Before starting the neural analyses, a cluster-based permutation test was conducted across all conditions during the baseline period (−200 to 0 ms) to confirm that the fixation cross displayed before stimulus onset did not result in consistent differences across conditions. This analysis was performed before the primary statistical analyses to confirm the comparability of baseline activity. The results showed no significant cluster differences (all *p* > 0.05), proving that visual processing of the fixation cross did not differentially affect baseline activity across conditions. Consequently, the same baseline interval was used for baseline correction in all subsequent studies.

ERP analysis was performed on the preprocessed EEG data using the Fieldtrip toolbox (version 20240110, http://www.fieldtriptoolbox.org/download, accessed on 10 March 2025) [46] in MATLAB 2024b (The MathWorks Inc., Natick, MA, USA). First, the data were filtered with a low-pass filter at 30 Hz to retain the frequencies of interest for event-related potential analysis, and baseline correction was applied using the interval from −200 to 0 ms. The early components of interest, P100 and N100, were quantified at selected electrode sites based on topographical maps, amplitude traces, and previous studies [47,48]. The P1 component was analyzed in the 90–160 ms window at positions Oz, O1, and O2 [47], while the N1 component was analyzed in the 100–160 ms window at Fz, Cz, and FCz [48]. Peaks were individually identified in the respective averaged data. Values were determined as the maximum amplitude (peak) within the defined time windows. For each subject and each condition (Alcohol, NeutralAlc, Food, NeutralFo), maximal (or minimal for N100) latencies and amplitudes were automatically extracted from the ERP signals at the defined electrodes by searching for peaks within the relevant time window.

#### 2.3.4. Ratings Data

After the posturographic and EEG recordings, participants moved on to the subjective evaluation phase, during which they rated visual stimuli previously presented under the same experimental conditions. A total of 208 images were displayed using E-Prime 3.0 stimulus software (Psychology Software Tools, Inc., Pittsburgh, PA, USA), each shown for 3 s. Following each image, participants answered six standardized questions assessing pleasantness, unpleasantness, approach desire, consumption desire, avoidance desire, and intensity, using a 9-point scale. Responses were entered via a computer keyboard, with no time limit, though participants were encouraged to respond promptly. The evaluation phase mirrored the structure of the experimental phase, consisting of eight sessions with 26 images each, interspersed with three-minute rest breaks between blocks.

### 2.4. Statistical Analysis

All statistical analyses were performed using R 4.4.3 software (R Core Team, Vienna, Austria) via the RStudio 2024.12.0 interface (RStudio, PBC, Boston, MA, USA). Postural and EEG Data were analyzed using a Type III ANOVA based on F-tests (Satterthwaite’s method). In the case of a significant main effect, post hoc comparisons were conducted using estimated marginal means (EMMeans), with Bonferroni correction for multiple comparisons. The comparisons of interest specifically focused on three main contrasts: Alc vs. NeutralAlc, Food vs. NeutralFo, and Alc vs. Food.

Since the ratings data did not follow a normal distribution (Shapiro–Wilk test) and homogeneity of variances was not met (Levene’s test), non-parametric tests were used. For within-group analyses (comparisons between conditions within the same group), paired Wilcoxon tests were applied. For between-group analyses (comparison between “Low” and “High”), Mann–Whitney tests (unpaired Wilcoxon) were used.

To assess the relationship between early neural responses and postural adjustments, Pearson’s correlation coefficients (r) were computed between ERP measures (P100 and N100 peak amplitudes and latencies) and anteroposterior center of pressure (COP-AP) displacement. These analyses were conducted separately for alcohol-related and food-related stimuli. Two postural metrics were considered: COP-AP displacement at 1 s post-stimulus onset, and mean COP-AP displacement across the entire 7 s trial. Statistical significance was set at *p* < 0.05.

Finally, Pearson correlation coefficients (r) were computed to examine the linear relationships between behavioral scores (AUDIT and DEBQ3) and both postural (COP-AP displacement) and electrophysiological (ERP amplitude and latency) measures. Correlations were performed separately for alcohol-related and food-related conditions. Statistical significance was set at *p* < 0.05.

## 3. Results

### 3.1. Psychometric Data

The psychometric data of the study groups are presented in Table 1. The sex distribution differs slightly between groups, with a majority of men in the “High” group and a majority of women in the “Low” group. Both groups are homogeneous in terms of age as well as scores on the depression (BDI-II) and anxiety (STAI) assessment questionnaires. As expected, marked differences are observed in AUDIT scores, indicating higher-risk alcohol consumption behaviors in the “High” group. Descriptive analysis also reveals slightly higher scores on the eating behavior assessment questionnaire (DEBQ) in this group. Finally, nicotine dependence, as measured by the Fagerström Test, is low across both groups.

### 3.2. Posturographic Data

**Figure 2:**
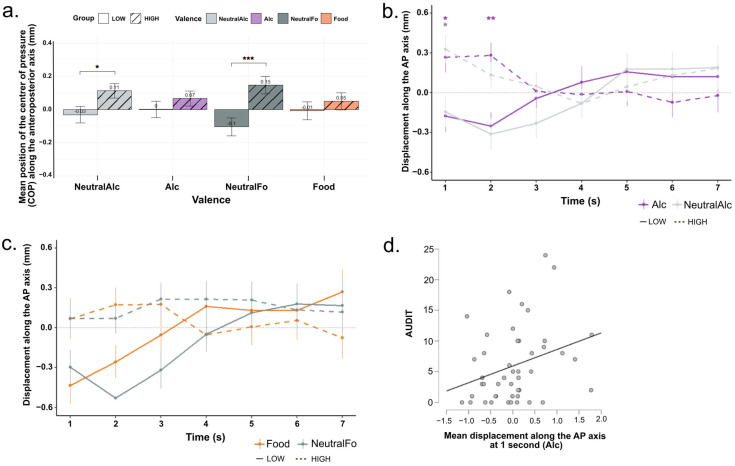
(**a**) Mean displacement and (**b**,**c**) time course over 7 s of the center of pressure along the anteroposterior axis (COP-AP) for the four stimulus conditions and the “Low” and “High” groups. Bars represent the mean for each condition, separated by alcohol consumption level (LOW: solid; HIGH: hatched/dotted). Error bars indicate the standard deviation around the mean. * *p* < 0.05, ** *p* < 0.01, *** *p* < 0.001 (**d**) Correlation between AUDIT scores and postural displacement (COP-AP) at 1 s after alcohol-related stimuli. Each gray dotrepresents a participant.

Figure 2a presents the mean displacement of the center of pressure along the anteroposterior axis (COP-AP) as a function of the four conditions (Alc, NeutralAlc, Food, NeutralFo) and the two participant groups defined by their AUDIT scores (“Low” and “High”). A repeated-measures ANOVA (Type III, with Greenhouse-Geisser correction) was performed on the COP-AP values. The analysis revealed a significant main effect of alcohol consumption level (F(1, 44) = 8.93, *p* = 0.005, η^2^ = 0.17), indicating an overall influence of alcohol consumption level on center of pressure displacement. In contrast, neither the main effect of valence (F(2.58, 113.62) = 0.11, *p* = 0.934, η^2^ = 0.002) nor the interaction between valence and consumption level (F(2.58, 113.62) = 2.38, *p* = 0.083, η^2^ = 0.05) reached significance. As shown in Figure 2a, the “High” group appears to exhibit greater anterior displacement compared to the “Low” group across all conditions. However, post hoc tests revealed that significant differences between groups were observed only for the NeutralAlc (*p* = 0.0371, d = 0.62) and NeutralFo (*p* < 0.001, d = 1.10) conditions.

To further examine postural dynamics, a second-by-second repeated-measures ANOVA (Type III, with Greenhouse–Geisser correction) was conducted on COP-AP values, considering Alcohol_Level (High vs. Low), Valence (Alc, NeutralAlc, Food, NeutralFo), and Time (Seconds 1–7) as factors (Figure 2b,c). The analysis revealed a significant main effect of Alcohol_Level (F(1, 44) = 8.93, *p* = 0.005, η^2^ = 0.008), indicating that overall, participants in the “High” group exhibited greater anterior displacement compared to the “Low” group. There was no significant main effect of Valence (F(2.58, 113.62) = 0.11, *p* = 0.934, η^2^ = 0.00014), nor a significant Alcohol_Level × Valence interaction (F(2.58, 113.62) = 2.38, *p* = 0.083, η^2^ = 0.003). A significant Alcohol_Level × Time interaction was observed (F(2.59, 113.62) = 3.39, *p* = 0.026, η^2^ = 0.03), suggesting that the effect of alcohol consumption level on postural displacement varied across the time course of stimulus presentation. As shown in Figure 2b, post hoc pairwise comparisons revealed that significant differences between the “High” and “Low” groups emerged as early as the first second for both the Alc (*p* = 0.027, d = 0.67) and NeutralAlc (*p* = 0.030, d = 1.02) conditions. At 2 s, the difference remained significant for the Alc condition (*p* = 0.009, d = 0.85) only. Specifically, participants in the “High” group exhibited a marked anterior (forward) displacement from the very first seconds, suggesting an automatic approach behavior regardless of stimulus valence (alcoholic or neutral). Conversely, participants in the “Low” group showed a posterior (backward) displacement at the same time points, indicating an early avoidance response. From the third second onward, the trajectories of the two groups converged, reflecting a gradual return to a similar center of pressure displacement levels. For the Food and NeutralFo conditions (Figure 2c), group differences did not reach statistical significance (all *p* > 0.05).

We examined correlations between postural responses and self-reported AUDIT (alcohol use) and DEBQ3 (external eating behavior) scores, focusing on the Alcohol and Food conditions (See Table A1). Among all the correlations tested, only one was found to be significant: a positive correlation between AUDIT scores and COP-AP displacement at 1 s post-stimulus onset in the Alcohol condition (r = 0.305, *p* = 0.04; Figure 2d). This suggests that individuals with higher levels of problematic alcohol use exhibit an enhanced early bodily approach tendency toward alcohol-related cues. No significant correlations were observed between AUDIT scores and mean COP-AP displacement over the 7-s post-stimulus period, indicating a specific association between alcohol use patterns and early automatic motor responses to alcohol stimuli. Additionally, no significant correlations were observed between DEBQ scores and postural responses in the food condition.

Overall, these results suggest the existence of automatic and differentiated postural responses depending on the level of alcohol consumption. These responses appear rapidly after stimulus presentation but are transient and limited to the very first seconds of visual exposure.

### 3.3. Subjective Ratings

**Figure 3:**
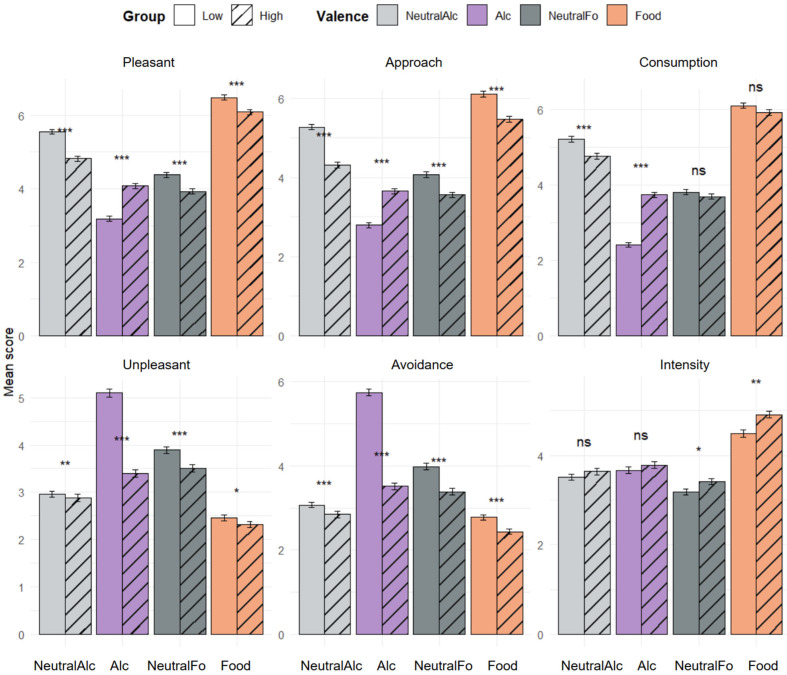
Mean subjective evaluation scores for the four stimulus conditions and the “Low” and “High” groups across six motivational and affective dimensions (Pleasure, Displeasure, Approach, Avoidance, Consumption, Intensity). Bars represent the mean scores on a scale from 1 to 9 for each condition, separated by alcohol consumption level (LOW: solid; HIGH: hatched). Error bars indicate the standard deviation around the mean. Asterisks indicate significant differences between the two groups: * *p* < 0.05, ** *p* < 0.01, *** *p* < 0.001, ns: not significant.

Figure 3 presents the results of the subjective evaluations. Statistical analysis, performed using the Wilcoxon signed-rank test, consistently revealed significant differences between the “Low” and “High” groups in the dimensions of Pleasant, Unpleasant, Approach, and Avoidance for each type of stimulus presented. The High group scored significantly higher than the Low group for the Alcohol condition across all these dimensions. Conversely, for the other conditions (NeutralAlc, Food, NeutralFo), scores were higher in the Low group. However, no significant group differences were observed in the Consumption dimension for food-related stimuli (Food and NeutralFo), nor in the Intensity dimension for alcohol-related stimuli (Alc and NeutralAlc).

Regarding comparisons between pairs of conditions, the analysis did not reveal any significant differences between Alcohol and NeutralAlc in the High group, regardless of the dimension evaluated. Similarly, no difference was observed between these two conditions in the Intensity dimension for the Low group. Overall, both groups assigned higher scores to images of appetitive food (Food) compared to neutral food images (NeutralFo) in the dimensions of Pleasure, Approach, Consumption, and Intensity. Conversely, neutral images elicited higher scores in the Displeasure and Avoidance dimensions. Finally, the Low group assigned higher scores to non-alcoholic beverages (NeutralAlc) than to alcoholic stimuli in the dimensions of Pleasure, Approach, and Consumption, and conversely in the dimensions of Displeasure and Avoidance, reflecting a more negative perception of alcohol in this group.

### 3.4. Neural Responses

**Figure 4:**
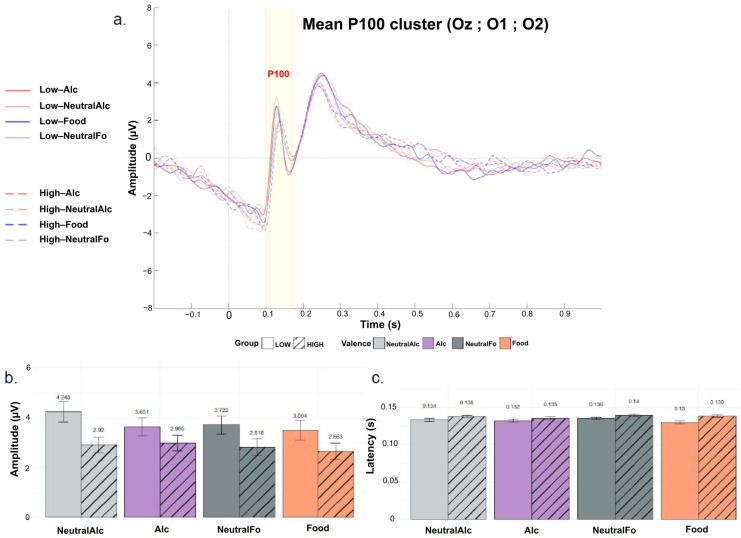
Grand-averaged event-related potentials (ERPs) for the P100 component, recorded over a cluster of occipital electrodes (Oz, O1, O2) for the four stimulus conditions and the “Low” and “High” groups. (**a**) Plot of overall mean amplitude; (**b**) Mean P100 peak latencies; (**c**) Mean P100 peak amplitudes. Curves are differentiated according to stimulus valence (Alc, NeutralAlc, Fo, nFo) and alcohol consumption level (LOW: solid lines, HIGH: dashed lines). Bars represent the mean for each condition, separated by alcohol consumption level (LOW: solid; HIGH: hatched). Error bars indicate the standard deviation around the mean.

Figure 4b illustrates the mean maximal P100 peak amplitudes for each condition. Statistical analysis revealed no significant main effect of Group (F(1, 44) = 1.42, *p* = 0.240, η^2^ = 0.03), Condition (F(3, 132) = 1.56, *p* = 0.202, η^2^ = 0.004), or Group × Condition interaction (F(3, 132) = 0.72, *p* = 0.541, η^2^ = 0.002). Although the differences did not reach statistical significance, P100 wave amplitudes tended to be higher in the “Low” group compared to the “High” group across all conditions. This pattern suggests a possible trend toward greater early neural responsiveness among low alcohol consumers, but this effect was not statistically confirmed in the present sample.

Figure 4c presents the mean P100 peak latencies for each condition. Statistical analysis revealed no significant main effect of Group (F(1, 44) = 2.32, *p* = 0.135, η^2^ = 0.04), Condition (F(3, 132) = 2.11, *p* = 0.102, η^2^ = 0.01), or Group × Condition interaction (F(3, 132) = 1.21, *p* = 0.310, η^2^ = 0.006). However, post hoc comparisons indicated that P100 latency was significantly shorter in the “Low” group compared to the “High” group, but only for appetitive food stimuli (*p* = 0.0401).

**Figure 5:**
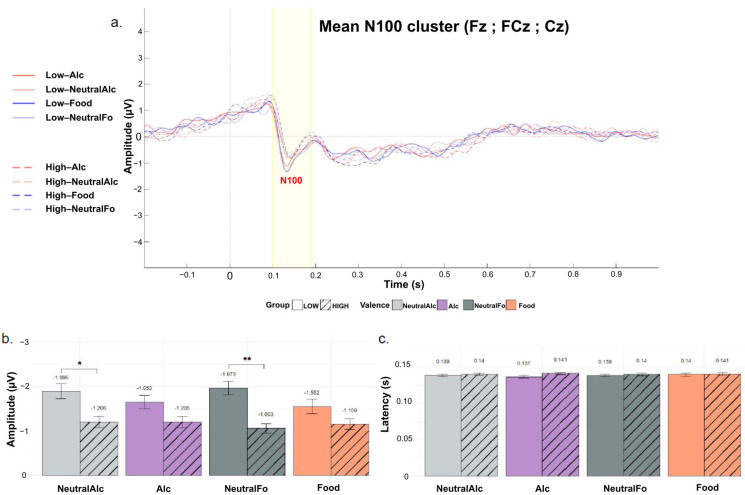
Grand-averaged event-related potentials (ERPs) for the N100 component, recorded over a cluster of fronto-central electrodes (Fz, FCz, Cz) for the four stimulus conditions and the “Low” and “High” groups. (**a**) Plot of overall mean amplitude; (**b**) Mean N100 peak latencies; (**c**) Mean N100 peak amplitudes. Curves are differentiated according to stimulus valence (Alc, NeutralAlc, Fo, nFo) and alcohol consumption level (LOW: solid lines, HIGH: dashed lines). Bars represent the mean for each condition, separated by alcohol consumption level (LOW: solid; HIGH: hatched). Error bars indicate the standard deviation around the mean. * *p* < 0.05, ** *p* < 0.01.

Figure 5b shows the mean maximal N100 peak amplitudes for each condition. Statistical analysis revealed a significant main effect of Group (F(1, 44) = 4.72, *p* = 0.035, η^2^ = 0.08), indicating that, on average, N100 amplitudes were more negative in the “Low” group compared to the “High” group. There was no significant main effect of Condition (F(3, 132) = 1.19, *p* = 0.316, η^2^ = 0.005) and no significant Group × Condition interaction (F(3, 132) = 2.15, *p* = 0.097, η^2^ = 0.01). Post hoc comparisons showed that for the Neutral Alcohol (*p* = 0.0467, d = 0.60) and Neutral Food (*p* = 0.0029, d = 0.93) conditions, N100 amplitudes were significantly more negative in the “Low” group than in the “High” group. These findings suggest that low alcohol consumers exhibit enhanced early neural responsiveness, particularly to neutral stimuli, as reflected by more negative N100 amplitudes in these conditions.

Figure 5c presents the mean N100 peak latencies for each condition. No significant modulation of N100 latency was observed as a function of Group (F(1, 44) = 0.48, *p* = 0.492, η^2^ = 0.007), Condition (F(3, 132) = 0.14, *p* = 0.938, η^2^ = 0.001), or Group × Condition interaction (F(3, 132) = 0.52, *p* = 0.667, η^2^ = 0.004).

**Figure 6:**
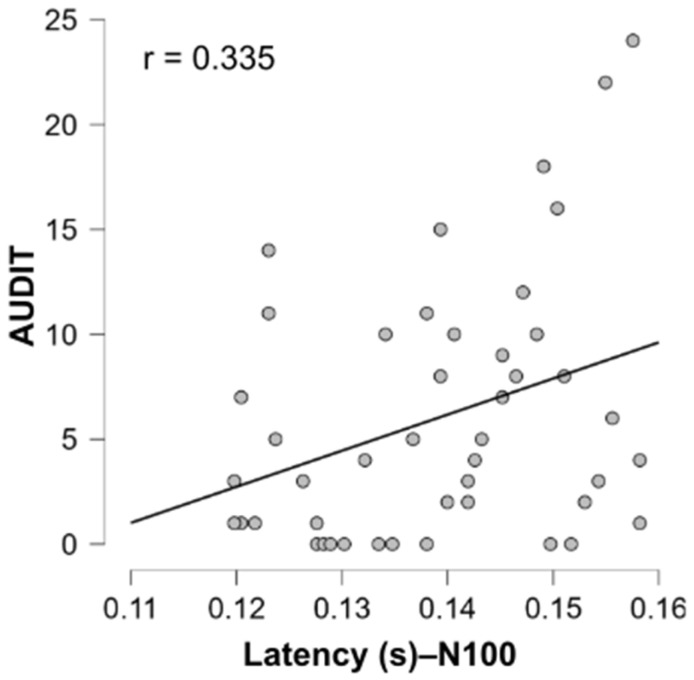
Positive correlations between N100 latency and AUDIT scores in response to alcohol-related stimuli. Each gray dot represents a participant.

Given that participants in the “High” group appeared to exhibit a tendency toward longer latencies and shorter amplitudes compared to the “Low” group (although this difference was not statistically significant), it was of relevance to test for a possible correlation between EEG responses and AUDIT score (See Table A1). We therefore tested for correlations between AUDIT scores and both amplitude and latency of the P100 and N100 components. Among all comparisons, only N100 latency showed a significant positive correlation with AUDIT scores (r = 0.335, *p* = 0.023), as illustrated in Figure 6. This suggests that individuals with higher AUDIT scores tend to exhibit slower early perceptual processing, indexed by delayed N100 responses. No other correlations were significant, including those between AUDIT scores and ERP amplitudes or P100 latency, suggesting that this effect may be specific to N100 latency. Similarly, no significant associations were identified between ERP measures and DEBQ3 scores for food-related stimuli (See Table A1).

Overall, participants with lower alcohol consumption showed a pattern of stronger and faster early neural responses (P100/N100), particularly to neutral stimuli, suggesting increased attentional engagement. In contrast, those with higher AUDIT scores tended to exhibit reduced amplitudes and delayed responses, with N100 latency positively associated with AUDIT scores, reflecting slower early perceptual processing in individuals with heavier alcohol use.

### 3.5. ERP–Posture Correlations

**Figure 7:**
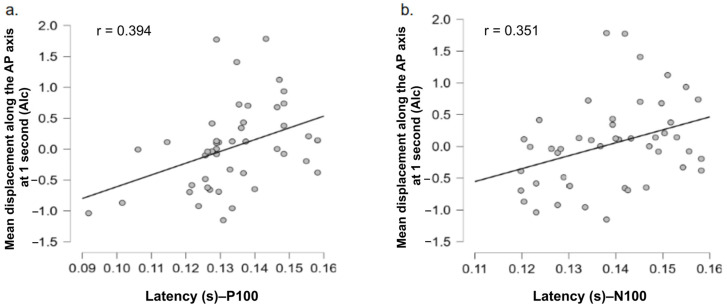
Positive correlations between the latency of (**a**) P100 and (**b**) N100 components and postural displacement (COP-AP) at 1 s following alcohol-related stimuli. Each gray dot represents a participant.

Table 2 presents the Pearson correlation coefficients between EEG measures (P100 and N100 peak amplitudes and latencies) and anteroposterior center of pressure displacement (COP-AP) in response to alcohol and food-related stimuli. For the P100 component, a significant positive correlation was observed between P100 latency and COP-AP displacement at 1 s following alcohol stimulus onset (r = 0.394, *p* = 0.007). This relationship is illustrated in Figure 7a, showing that longer P100 latencies are associated with greater forward body displacement. No significant correlation was found between P100 latencies and mean center of pressure displacement over 7 s. For the N100 component (Figure 7b), a significant positive correlation was also found between N100 latency and COP-AP displacement at 1 s in response to alcohol stimuli (r = 0.351, *p* = 0.017). This suggests that a longer N100 latency is likewise associated with greater anterior body displacement in this condition. As with P100, no significant correlation was observed between N100 latencies and mean center of pressure displacement over 7 s.

No other significant correlations were found between wave amplitudes and center of pressure displacement for either the P100 or N100 components (Table 2). Similarly, no significant associations were identified between ERP measures and postural displacement for food-related stimuli (Table 2).

## 4. Discussion

### 4.1. Subjective Responses

Regarding the subjective data (Figure 3), the results are consistent with our hypothesis for alcohol-related stimuli. Compared to the “Low” group, the “High” group assigned higher scores to alcoholic stimuli in the dimensions of Pleasure, Approach, and Consumption. In contrast, the “Low” group showed a clear preference for non-alcoholic beverages and a more negative perception of alcoholic beverages. However, no difference was observed between Alcohol and NeutralAlc in the “High” group. This lack of distinction may reflect habituation or normalization of alcohol-related content among regular consumers. Another plausible explanation is that the images selected did not match participants’ personal preferences, thereby limiting their sensitivity to the images presented. Finally, as expected, both groups preferred images of appetitive food over neutral images, confirming the well-differentiated emotional valence of the food stimuli.

### 4.2. Postural Responses

One of the objectives of this study was to analyze postural responses to visual stimuli related to alcohol and food. The results (Figure 2) reveal that, while no significant differences were found between stimulus types, clear differences in anteroposterior center of pressure displacement (COP-AP) emerge between participants with “Low” and “High” AUDIT scores. Specifically, descriptive analyses showed a general tendency toward greater anterior displacement in the “High” group across all conditions. However, statistical differences between groups were significant only for the neutral conditions, with no significant effects observed in the motivationally salient conditions (Alcohol and Food).

To explore the temporal dynamics of these postural responses, a second-by-second analysis was conducted. This study shows that participants with higher AUDIT scores exhibit anterior displacement of the center of pressure (COP) as early as the first second following exposure to alcohol-related images. This forward orientation may be interpreted as a marker of automatic approach behavior, as described in the literature [16,17]. Noël with colleagues demonstrated that individuals with AUD display distinct body movements oriented toward alcohol-related cues [12]. In our study, although participants were not clinically dependent, a high AUDIT score alone appears sufficient to trigger a rapid and automatic bodily orientation toward alcohol cues. In contrast, participants in the “Low” group showed posterior displacement under the same conditions, reflecting an early avoidance response. This pattern aligns with subjective reports and may reflect automatic disengagement from stimuli perceived as irrelevant or potentially unpleasant, possibly linked to a more neutral or aversive internal representation of alcohol [7,8,9]. Regarding food-related stimuli, the second-by-second analysis showed a similar pattern, but no statistically reliable differences were observed for either Food or NeutralFo. Notably, the subjective ratings paradoxically indicated stronger approach motivation among “Low” participants compared to the “High” group, particularly for appetitive foods. This dissociation between subjective and postural responses may reflect a more complex or delayed motivational engagement with food stimuli.

Furthermore, a significant correlation was found between AUDIT scores and COP-AP displacement at 1 s post-stimulus onset in the Alcohol condition (Figure 2d), reinforcing the notion that higher alcohol use is associated with an early bodily approach tendency. This correlation was not observed for other conditions, suggesting a specific link between alcohol consumption patterns and automatic motor responses to alcohol cues.

Collectively, our results highlight the influence of consumption habits in shaping the temporal dynamics of postural responses. These findings indicate that early postural responses occurring within the first seconds of stimulus presentation may serve as sensitive markers of underlying motivational processes. This suggests a potential utility for using postural markers to assess behavioral vulnerability in at-risk populations.

It should also be noted that the significant differences observed in neutral conditions (NeutralAlc and NeutralFo) emerged between Low and High AUDIT groups. Since the AUDIT is a validated behavioral questionnaire, these group effects are anchored in well-established behavioral differences in alcohol consumption. However, because no direct subjective validation of the neutrality of these stimuli was collected, the motivational interpretation of these postural differences should be considered with caution. Future studies would benefit from combining posturographic measures with explicit ratings of stimulus neutrality in order to refine the interpretation of such effects.

### 4.3. ERP Responses

Beyond the motor responses observed through posture, it is also relevant to examine early neurophysiological correlates associated with stimulus perception, which may reveal automatic motivational processes [49].

In the present study (Figure 4 and Figure 5), descriptive data indicate a tendency toward higher P100 and N100 amplitudes in participants from the “Low” group, regardless of stimulus valence, suggesting stronger detection and attentional engagement among low consumers. In contrast, participants in the “High” group tend to exhibit reduced amplitudes and slightly longer latencies for these components, independent of image type. This slowing of perceptual processing may reflect decreased vigilance or impaired sensory efficiency. These observations are consistent with the findings of Maurage with colleagues who demonstrated that young adults with binge drinking behaviors show reduced ERP amplitudes and increased latencies when presented with emotional faces [50]. Regarding the N100 component, group differences were more pronounced. Participants in the “Low” group exhibited significantly greater N100 amplitudes than those in the “High” group for neutral stimuli specifically. Some studies showed that early components such as the N100 are sensitive to the automatic orientation of attention toward stimuli deemed relevant [33,51]. In the present context, where neutral food items elicit less affective or hedonic interest, a greater N100 amplitude may reflect increased attentional allocation to process content perceived as less emotionally engaging, contrary to what is generally reported in the literature [33,34,51].

This apparent discrepancy with incentive-sensitization accounts suggests a dissociation between amplitude and latency effects. While amplitude did not consistently vary with stimulus valence, latency differences were more robust and specific to alcohol cues, with High consumers showing delayed N100 processing. This pattern indicates that latency may represent a more reliable index of perceptual encoding differences related to consumption habits, whereas amplitude in Low consumers might be preferentially recruited for ambiguous or weakly motivational stimuli.

Although the group differences in latency and amplitude did not reach statistical significance, these trends are noteworthy, particularly in light of the significant positive correlation observed between N100 latency and AUDIT scores (Figure 6). This suggests that even subtle variations in early perceptual processing may be meaningfully related to individual differences in alcohol use.

Taken together, these results highlight the influence of consumption levels on early perceptual processing of visual stimuli. Participants with low alcohol consumption (“Low”) exhibited more pronounced and differentiated neural reactivity, suggesting greater attentional engagement with the stimuli. In contrast, frequent consumers (“High”) showed attenuated and less discriminative responses, indicating possible habituation or reduced vigilance. These observations are consistent with models proposing that preferences and consumption habits influence attentional salience from the earliest stages of sensory processing.

It should be noted that, unlike posturographic data which were analyzed on a second-by-second basis, EEG analyses were restricted to early ERP components (P100, N100) defined in narrow temporal windows. This methodological asymmetry limits the possibility of establishing a strictly parallel temporal dynamic between the two modalities. Future studies using time–frequency analyses or dynamic multimodal modeling could provide a more continuous characterization of perceptual–motor coupling. Although our study included a relatively large sample for posturographic research (N = 46), which strengthens the robustness of behavioral outcomes, post-hoc sensitivity analyses indicate that it was mainly powered to detect medium-to-large effects, and small ERP amplitude differences may therefore have been missed.

### 4.4. EEG–Posture Correlations

The analysis of correlations between EEG and postural data (Figure 7) provides insight into the functional link between early neural activity and automatic bodily responses. This multimodal approach aims to better understand how early perceptual signals recorded by EEG (P100, N100) may be associated with approach or avoidance body movements, particularly in the context of highly motivational stimuli such as alcohol.

For both the P100 and N100 components, significant positive correlations were observed between their latency and postural displacement at 1 s after the presentation of alcohol-related images. In other words, longer latencies are associated with forward sway, while shorter latencies correspond to a postural withdrawal, consistent with approach or avoidance dynamics. No significant correlation was observed between latencies and mean postural displacement over 7 s, confirming that these relationships pertain to early, transient responses, in line with the temporal effects observed in the postural analysis. Moreover, no significant correlation was found between ERP component amplitudes (P100, N100) and postural displacement, nor for food-related stimuli. This may be explained by the lower motivational relevance of food content, or by greater individual variability in their affective perception. Gable and Harmon-Jones observed that affective states associated with approach motivation could amplify the amplitude of these components, suggesting that the emotional and motivational context plays a crucial modulatory role [52].

These results suggest that the latencies of early ERP components are functionally linked to postural responses in the context of alcohol, and that this link is potentially related to alcohol consumption habits. Taken together, these findings highlight the value of an integrative EEG–posture approach for studying the early mechanisms underlying automatic motivational behaviors. Although significant correlations were observed between ERP latencies and postural adjustments, their effect sizes were modest (around r = 0.35). In the context of cognitive and behavioral neuroscience, such values are considered small-to-moderate but remain meaningful given the complexity of perceptual–motor interactions. These findings should therefore be interpreted as preliminary evidence of coupling rather than as strong proof of a direct link. Future studies with larger samples will be needed to confirm the robustness and generalizability of these associations.

It should be noted that our study did not aim to propose a formal mechanistic model integrating EEG and posturographic signals. Rather, our approach was exploratory, motivated by the fact that very few studies have so far combined these two modalities within the same paradigm. In this context, our results primarily provide proof of concept, showing that early perceptual markers (P100, N100) and motor responses can be jointly recorded and are functionally associated. Future work will be needed to develop more formal frameworks, for example computational or dynamical models, that could account for the mechanisms underlying this perceptual–motor coupling. Another limitation is that the “High” group included a higher proportion of males and exhibited elevated external eating scores compared to the “Low” group. Both sex and eating traits have been shown to modulate attentional and postural responses to appetitive stimuli, and these imbalances therefore represent potential confounds. Although group allocation was based solely on AUDIT scores, a validated behavioral measure of alcohol use, these additional differences may have influenced our results. Our findings should thus be interpreted with caution, and future studies are needed to better control for sex and eating-related characteristics when investigating alcohol-related reactivity.

For future research, it would be valuable to explore more immersive contexts (e.g., virtual reality) or to include clinical populations, in order to better understand the interaction between perceptual and motor responses in risk behaviors. It would also be pertinent to select stimuli based on participants’ subjective ratings to better account for interindividual differences in affective perception of the presented content.

## 5. Conclusions

This study highlights differences between individuals with low and high alcohol consumption in their reactivity to visual stimuli, both in postural and electrophysiological terms. Participants with a high AUDIT score exhibited a tendency toward longer perceptual latencies and more pronounced approach-related bodily responses to alcohol, while “Low” participants demonstrated faster attentional engagement and avoidance reactions. These results suggest an early coupling between perception and action, modulated by consumption habits.

From both theoretical and practical perspectives, our findings suggest that alcohol-related cues influence both early neural processing and postural adjustments, underscoring the embodied nature of motivational salience. This suggests that perceptual–motor coupling contributes to attentional biases from the earliest stages of information processing. Practically, the integration of EEG and posturography offers a promising multimodal framework to identify subtle markers of risky drinking tendencies, with potential relevance for prevention and early detection.

However, several limitations should be considered when interpreting these findings. Among the study’s limitations, the choice of images, although standardized, may not have sufficiently accounted for individual preferences, particularly for food stimuli. Additionally, the sample, consisting mainly of young adults, limits the generalizability to other age groups.

## Figures and Tables

**Table 1:** Means and standard deviations (in parentheses) for the “Low” and “High” groups on the AUDIT, BDI-II, STAI-T, DEBQ, and Fagerström Test.

	Low (*n* = 24)	High (*n* = 22)
**Sex** (**♀:♂**)	15:9	8:14
**Age (years)**		
Women	24.87 (8.62)	27 (11.71)
Men	24.33 (2.92)	25.50 (5.75)
**AUDIT**	1.46 (1.47)	10.95 (5.30)
**BDI-II**	8.83 (6.87)	9.27 (7.75)
**STAI TRAIT**	39.17 (8.15)	39.45 (11.09)
**DEBQ Restrained eating**	1.93 (0.69)	2.40 (0.92)
**DEBQ Emotional eating**	2.04 (0.69)	2.25 (0.91)
**DEBQ External eating**	2.93 (0.63)	3.23 (0.62)
**Fagerström Test**	0.79 (1.98)	0.64 (1.71)

AUDIT: Alcohol Use Disorders Identification Test. BDI: Beck Depression Inventory-II. STAI: Trait Anxiety Inventory. DEBQ: Dutch Eating Behavior Questionnaire.

**Table 2:** Pearson’s correlation coefficients (r) and *p*-values between P100/N100 components and anteroposterior center of pressure displacement in response to alcohol and food related stimuli.

		Alcohol-Related Stimuli	Food-Related Stimuli
**P100**	**Amplitude—meanCOP-AP**	r = −0.147 *p* = 0.329	r = 0.079 *p* = 0.600
	**Amplitude—COP-AP (1 s)**	r = −0.061 *p* = 0.688	r = −0.197 *p* = 0.189
	**Latency—meanCOP-AP**	r = 0.040 *p* = 0.792	r = 0.019 *p* = 0.902
	**Latency—COP-AP (1 s)**	r = 0.394 ** *p* = 0.007	r = 0.144 *p* = 0.341
**N100**	**Amplitude—meanCOP-AP**	r = −0.004 *p* = 0.980	r = −0.087 *p* = 0.565
	**Amplitude—COP-AP (1 s)**	r = 0.124 *p* = 0.411	r = 0.118 *p* = 0.435
	**Latency—meanCOP-AP**	r = 0.116 *p* = 0.441	r = 0.130 *p* = 0.389
	**Latency—COP-AP (1 s)**	r = 0.351 * *p* = 0.017	r = 0.089 *p* = 0.556

Amplitude: Maximal peak amplitudes of the P100 or N100 component. Latency: Peak latency of the P100 or N100 component. meanCOP-AP: Mean displacement of the center of pressure along the anteroposterior axis. COP-AP (1 s): Displacement of the center of pressure along the anteroposterior axis at 1 s. * *p* < 0.05, ** *p* < 0.01.

## Data Availability

The data presented in this study are available on request from the corresponding author.

## Supplementary Materials

The following supporting information can be downloaded at: https://www.mdpi.com/article/10.3390/biology14091172/s1, SOM Stimuli databases selection procedure (reference [53] is cited in the supplementary materials).

## Appendix A

**Table A1:** Scores and EEG/postural measures in response to alcohol and food related stimuli.

	Stimuli	Alcohol	Food
**COP-AP**	**AUDIT—meanCOP-AP**	r = 0.174 *p* = 0.147	-
	**AUDIT—COP-AP (1 s)**	**r = 0.305 * *p* = 0.04**	-
	**DEBQ3—meanCOP-APy**	-	r = 0.092 *p* = 0.543
	**DEBQ3—COP-AP (1 s)**	-	r = 0.099 *p* = 0.513
**P100**	**AUDIT—Amplitude**	r = −0.159 *p* = 0.291	-
	**AUDIT—Latency**	r = 0.183 *p* = 0.224	-
	**DEBQ3—Amplitude**	-	r = 0.070 *p* = 0.643
	**DEBQ3—Latency**	-	r = 0.119 *p* = 0.441
**N100**	**AUDIT—Amplitude**	r = 0.100 *p* = 0.507	-
	**AUDIT—Latency**	r = 0.335 *p* = 0.023	-
	**DEBQ3—Amplitude**	-	r = −0.127 *p* = 0.400
	**DEBQ3—Latency**	-	r = 0.134 *p* = 0.374

Amplitude: Maximal peak amplitudes of the P100 or N100 component. Latency: Peak latency of the P100 or N100 component. meanCOP-AP: Mean displacement of the center of pressure along the anteroposterior axis. COP-AP (1 s): Displacement of the center of pressure along the anteroposterior axis at 1 s. AUDIT: Alcohol Use Disorders Identification Test. DEBQ3: Dutch Eating Behavior Questionnaire—External eating. -: No value (not applicable). * *p* < 0.05.
